# Replacement of dietary carbohydrate with protein increases fat mass and reduces hepatic triglyceride synthesis and content in female obese Zucker rats

**DOI:** 10.14814/phy2.15885

**Published:** 2023-11-30

**Authors:** M. Alan Dawson, Stephen R. Hennigar, Mahalakshmi Shankaran, Alyssa M. Kelley, Bradley J. Anderson, Edna Nyangau, Tyler J. Field, William J. Evans, Marc K. Hellerstein, James P. McClung, Stefan M. Pasiakos, Claire E. Berryman

**Affiliations:** ^1^ Department of Nutrition and Integrative Physiology Florida State University Tallahassee Florida USA; ^2^ Military Nutrition Division US Army Research Institute of Environmental Medicine Natick Massachusetts USA; ^3^ Oak Ridge Institute for Science and Education Belcamp Maryland USA; ^4^ Pennington Biomedical Research Center Louisiana State University Baton Rouge Louisiana USA; ^5^ Department of Nutritional Sciences and Toxicology University of California Berkeley California USA; ^6^ Military Performance Division US Army Research Institute of Environmental Medicine Natick Massachusetts USA

**Keywords:** cholesterol, fat/fatty acids, lipid flux, lipid metabolism

## Abstract

Previous studies have demonstrated both energy restriction (ER) and higher protein (HP), lower carbohydrate (LC) diets downregulate hepatic de novo lipogenesis. Little is known about the independent and combined impact of ER and HP/LC diets on tissue‐specific lipid kinetics in leptin receptor‐deficient, obese rodents. This study investigated the effects of ER and dietary macronutrient content on body composition; hepatic, subcutaneous adipose tissue (SAT), and visceral AT (VAT) lipid metabolic flux (^2^H_2_O‐labeling); and blood and liver measures of cardiometabolic health in six‐week‐old female obese Zucker rats (Lepr^fa+/fa+^). Animals were randomized to a 10‐week feeding intervention: ad libitum (AL)‐HC/LP (76% carbohydrate/15% protein), AL‐HP/LC (35% protein/56% carbohydrate), ER‐HC/LP, or ER‐HP/LC. ER groups consumed 60% of the feed consumed by AL. AL gained more fat mass than ER (P‐energy = 0.012) and HP/LC gained more fat mass than HC/LP (P‐diet = 0.025). Hepatic triglyceride (TG) concentrations (P‐interaction = 0.0091) and absolute hepatic TG synthesis (P‐interaction = 0.012) were lower in ER‐HP/LC versus ER‐HC/LP. ER had increased hepatic, SAT, and VAT de novo cholesterol fractional synthesis, absolute hepatic cholesterol synthesis, and serum cholesterol (P‐energy≤0.0035). A HP/LC diet, independent of energy intake, led to greater gains in fat mass. A HP/LC diet, in the context of ER, led to reductions in absolute hepatic TG synthesis and TG content. However, ER worsened cholesterol metabolism. Increased adipose tissue TG retention with the HP/LC diet may reflect improved lipid storage capacity and be beneficial in this genetic model of obesity.

## 1 INTRODUCTION

The prevalence of obesity and its associated metabolic complications have increased substantially over the last 20 years, increasing from 30.5% in 1999–2000 to 42.4% in 2017–2018 (Hales et al., 2020). Obesity is associated with a constellation of metabolic abnormalities, including insulin resistance (Kahn & Flier, 2000), type 2 diabetes (Bhupathiraju & Hu, 2016), and non‐alcoholic fatty liver disease (NAFLD) (Samji et al., 2019). Numerous studies have revealed that obesity is associated with changes in adipose tissue (AT), where subcutaneous AT (SAT) no longer effectively stores triglyceride (TG). The impeded ability to store TG in AT, either by decreased TG synthesis or increased lipolysis, is associated with greater lipid mobilization, ectopic lipid deposition, and insulin resistance (Longo et al., 2019; Nouws et al., 2019; Ravussin & Smith, 2002; Reilly & Saltiel, 2017; Rutkowski et al., 2015; White & Ravussin, 2019). Furthermore, conditions such as obesity‐induced insulin resistance and the metabolic syndrome are associated with decreased SAT TG synthesis and reduced SAT and VAT de novo lipogenesis (DNL) (Allister et al., 2015; Tuvdendorj et al., 2013). However, obesity increases the expression of genes involved in hepatic DNL, which have been associated with poor metabolic outcomes, including NAFLD, which is characterized by an accumulation of TG and free cholesterol in the liver (Eissing et al., 2013; Kerr & Davidson, 2012; Min et al., 2012). AT dysfunction, in combination with insulin resistance and elevated rates of hepatic DNL and cholesterol synthesis, can contribute to the development of NAFLD (Godoy‐Matos et al., 2020; Sanders & Griffin, 2016).

Lifestyle changes resulting in weight loss remain the cornerstone of treatment for obesity and NAFLD (Chalasani et al., 2018). Energy restricted diets that lead to a 3%–10% weight loss are associated with reductions in intrahepatic TG, decreased hepatic DNL, and improved insulin sensitivity in participants with obesity and NAFLD (Smith et al., 2020; Vilar‐Gomez et al., 2015). Excess dietary carbohydrate increases hepatic DNL rates (Chiu et al., 2018), although both low‐fat and low‐carbohydrate diets have been shown to reduce intrahepatic TG and improve NAFLD in patients with obesity (Parra‐Vargas et al., 2020). Energy restriction (ER) and reduced dietary carbohydrate intake have been shown to decrease hepatic DNL and intrahepatic TG content in normal weight rats and humans (Margolis et al., 2016; Schwarz et al., 1995). However, the combined effects of ER and altered protein and carbohydrate intake on hepatic, SAT, and VAT lipid kinetics in an obese rodent model are unknown.

The objectives of this study were to determine the independent and combined effects in an obese Zucker rat model of 10‐week ER and altered dietary protein and carbohydrate content on, (1) TG synthesis and DNL in SAT, VAT, and hepatic tissue, (2) cholesterol de novo synthesis in SAT, VAT, and hepatic tissue, (3) serum TG, free fatty acid, and cholesterol concentrations, and (4) hepatic TG and cholesterol concentrations. The Zucker rat has a mutation in the gene encoding the leptin receptor and as a result displays hyperphagia and obesity (Bray, 1977). We hypothesized that ER would lead to lower hepatic TG synthesis and greater SAT and VAT TG synthesis and that both ER and high dietary protein, low carbohydrate intake would result in lower hepatic DNL.

## 2 MATERIALS AND METHODS

### 2.1 Animals and study design

Six‐week‐old, female Zucker (Lepr^fa+/fa+^) rats (*n* = 46, Charles River Laboratories, Cambridge, MA) were housed in individual polycarbonate cages with a 12‐h light/dark cycle (dark cycle: 1900‐0700) in temperature and humidity controlled rooms (23 ± 2°C/40%–50%) in an approved animal care facility during the 10‐weeks intervention, as previously reported (Varanoske et al., 2021). Animals had ad libitum access to water and AIN‐93M purified diet (Reeves et al., 1993) during a 2‐week acclimation period. Following the acclimation period, animals were randomized into one of four diet intervention groups matched for body mass (*n* = 11‐12/group): ad libitum‐high carbohydrate, low protein (AL‐HC/LP), ad libitum‐high protein, low carbohydrate (AL‐HP/LC), energy restricted‐high carbohydrate, low protein (ER‐HC/LP), or energy‐restricted‐high protein, low carbohydrate (ER‐HP/LC). Lipid kinetic measures were a secondary outcome in a study investigating the impact of ER and high dietary protein intake on mineral absorption and musculoskeletal health. The companion paper reported protein synthesis measures in response to ER and dietary protein intake (Varanoske et al., 2021). The study protocol was approved by the US Army Research Institute of Environmental Medicine (USARIEM) Animal Care and Use Committee.

### 2.2 Body composition

Longitudinal changes in body composition were assessed in a subset of animals (*n* = 4/group) using nuclear magnetic resonance imagining (NMRI) at baseline and after 10 weeks on the experimental diets. Animals were briefly placed in a well‐ventilated Plexiglass tube and placed into a specialized NMRI machine for rodents (Minispec LF90II, Bruker‐BioSpin, Inc.). Data were analyzed by Minispec software (Bruker‐Biospin, Inc.) and used to determine fat mass.

### 2.3 Diet intervention

Diets were formulated by Research Diets, Inc. (New Brunswick, NJ). The AL‐HC/LP and AL‐HP/LC groups were given ad libitum access to standard AIN‐93M diet (15% protein and 76% carbohydrate) or a modified AIN‐93M diet containing 35% protein (140 g casein/kg diet vs. 332.5 g casein/kg diet) and 56% carbohydrate, respectively (Table 1). The ER‐HC/LP and ER‐HP/LC groups were fed 60% of the feed intake consumed by the AL control group (i.e., 40% ER). Feed intake of the AL groups was measured daily, and ER groups were provided with 60% of the feed by weight consumed by AL rats on the previous day. ER rats were fed each day between 0800 and 0900. Water intake was measured daily.

**TABLE 1:** Diet composition.^a^ ^,^ ^b^ ^,^ ^c^

	AL‐HC/LP	AL‐HP/LC	ER‐HC/LP	ER‐HP/LC
Macronutrients				
Protein, % kcal	15	35	15	35
Carbohydrate, % kcal	76	56	76	56
Fat, % kcal	9	9	9	9
Energy, kcal/g	3.8	3.8	3.8	3.8
Ingredients				
Casein, 30 Mesh, g	140	333	140	333
L‐cystine, g	1.8	1.8	1.8	1.8
Corn starch, g	496	303	492	299
Maltodextrin 10, g	125	125	125	125
Sucrose, g	100	100	100	100
Cellulose, BW200, g	50	50	50	50
Soybean oil, g	40	40	40	40
Mineral mix S10022M, g	35	35	58	58
Vitamin mix V10001, g	10	10	17	17
Choline bitartrate, g	2.5	2.5	2.5	2.5
Nutrient, unit/100 g				
Calories, kcal	373	374	363	365
Calories from fat, kcal	37	38	33	37
Saturated fatty acids, g	0.7	0.8	0.6	0.8
Unsaturated fatty acids, g	3.2	3.2	2.9	3.1
Monounsaturated fatty acids, g	0.9	0.9	0.8	0.9
Polyunsaturated fatty acids, g	2.3	2.3	2.0	2.2
Trans fatty acids, g	0.05	0.05	0.04	0.05
Cholesterol, mg	3.0	7.2	3.4	7.6
Fiber, g	4.9	4.8	4.6	4.7
Sugar, g	12	12	13	13

^a^ Table reproduced with permission from (Varanoske et al., 2021).

^b^ Diets were based on American Institute of Nutrition (AIN)‐93M, formulated by Research Diets, Inc., and analyzed by Eurofins Food Integrity & Innovation.

^c^ Energy restricted groups (ER‐HC/LP and ER‐HP/LC) were fed 60% of the feed intake consumed by the AL‐HC/LP group the previous week.

Abbreviations: AL‐HC/LP, ad libitum high carbohydrate, low protein; AL‐HP/LC, ad libitum high protein, low carbohydrate; ER‐HC/LP, energy restricted high carbohydrate, low protein; ER‐HP/LC, energy restricted high protein, low carbohydrate.

### 2.4 Lipid kinetic measurements

TG synthesis, DNL, and de novo cholesterol synthesis were measured in inguinal and epididymal fat pads as measures of SAT and VAT, respectively, and hepatic tissue through the use of ^2^H_2_O labeling combined with mass isotopomer distribution analysis, as described previously (Hellerstein & Neese, 1992; Lee et al., 1994; Turner et al., 2003). All animals received a priming dose of 99% deuterated water (^2^H_2_O; Sigma‐Aldrich, St. Louis, MO) at 0.035 mL/g body weight via intraperitoneal injection at Week 9 of the 10‐week study. Isotopic equilibrium was maintained by providing rats isotopically‐enriched (8% to maintain 5% ^2^H‐enrichment) water ad libitum throughout the remainder of the intervention (Pierce et al., 2016). De novo cholesterol synthesis, TG synthesis and DNL were measured in liver, SAT and VAT using methods adapted from those published previously (Shankaran et al., 2017; Turner et al., 2003; Turner et al., 2007). Briefly, lipid was extracted from ~20 mg tissue into 2:1 chloroform: methanol and prepared for thin layer chromatography to separate cholesterol and TGs on silica coated TLC plates (Sorbent Technologies, Inc., catalog no: 2315126C). The lipids were visualized with rhodamine solution (Sigma‐Aldrich, catalog no: R4127), scraped off the TLC plate and extracted into chloroform prior to hydrolysis and derivatization. Cholesterol samples were hydrolyzed with methanolic 3 N HCl for 1 h at 55°C and derivatized with acetyl chloride for analysis by GC–MS. TG samples were also incubated with methanolic 3 N HCl for 1 h at 55°C to effectively hydrolyze all complex lipids and convert fatty acids into fatty acid methyl esters (FAMEs), which were separated from glycerol by Folch extraction. The fatty acid methyl esters are separated by gas chromatography prior to analysis of isotopic enrichment by mass spectrometry (Turner et al., 2003). The fatty acid methyl esters in the organic layer were dried under nitrogen and resuspended in 1 mL heptane for GC–MS analysis. The glycerol fraction was dried, derivatized with 2:1 acetic anhydride: pyridine to the glycerol‐triacetate derivative, dried and re‐suspended in ethyl acetate for GC–MS analysis as described previously (Smith et al., 2020; Turner et al., 2003; Turner et al., 2007).

GC–MS analyses for measuring isotopic enrichments of cholesteryl acetate, FAMEs and glycerol triacetate were performed on an Agilent Technologies (Agilent, Palo Alto, CA) GC6890 equipped with a 5973 mass detector operated in selected ion‐monitoring mode. The cholesterol acetate derivative was analyzed using a DB‐17MS column (30 m × 0.25 mm × 0.25 μm) and the selected ions corresponding to M0 & M1 mass isotopomers for cholesterol were m/z 368 and 369. Glycerol triacetate was analyzed using a DB‐225 fused silica column, monitoring mass‐to‐charge ratios (m/z) 159, 160, and 161 (M0, M1, and M2, respectively). FAMEs were analyzed with a DB‐225 fused‐silica column under isothermal conditions at 200°C. For methyl palmitate, the molecular anion and its isotopes (m/z 270, 271, and 272 representing M0, M1, and M2) were quantified under the selected ion‐monitoring mode.

The precursor enrichment used for calculating the fractional synthesis of all the analytes was determined by MIDA of excess M2/M1 in liver palmitate. The fractional synthesis (f) was calculated based on the precursor‐product, or rise‐to‐plateau approach, *f* = EM1/A∞, where EM1 represents the mass + 1‐labeled species in excess of natural abundance, and A∞ represents the theoretical plateau or asymptotic value for fully labeled moiety. The theoretical plateau or asymptotic value A∞ during ^2^H_2_O labeling was determined by MIDA of the combinatorial labeling pattern based on precursor ^2^H–enrichment, as described elsewhere (Hellerstein & Neese, 1992). The fractional synthesis of palmitate in adipose TG underestimates the contribution of DNL to newly synthesized TG because new DNL is diluted by pre‐existing TG stores (Smith et al., 2020; Turner et al., 2003). Accordingly, the ratio of fractional DNL to fractional TG synthesis was used as the true fractional contribution of DNL to new TG‐palmitate.

Furthermore, estimates of absolute hepatic TG synthesis and cholesterol synthesis were determined by multiplying hepatic concentrations by hepatic fractional synthesis rates:

$$\begin{array}{c} \text{Absolute hepatic}\;\mathrm{TG}\;\text{synthesis}\\ =\mathrm{TG}\;{\text{concentration}}^{*}\text{fractional}\;\mathrm{TG}\;\text{synthesis}. \end{array}$$

$$\begin{array}{c} \text{Absolute hepatic cholesterol synthesis}\\ =\text{cholesterol}\;{\text{concentration}}^{*}\text{fractional cholesterol synthesis}. \end{array}$$

### 2.5 Euthanasia

At the end of the 10‐week study, rats were euthanized by CO_2_ asphyxiation after an overnight fast. Energy‐restricted animals had not eaten since the previous morning (i.e., ~24 h without food) compared to the ad libitum animals who were fasted overnight (i.e., ~12 h without food). Following CO_2_ asphyxiation, blood was collected by cardiac puncture, processed, and frozen at −80°C until analysis. Liver and AT (inguinal and epididymal AT pads) were extracted, snap frozen in liquid nitrogen, and subsequently stored at −80°C until analysis.

### 2.6 Metabolic measurements

Serum TG (catalog no: ab178780), cholesterol (catalog no: ab65359), and free fatty acid (catalog no: ab65341) concentrations and liver TG (catalog no: ab178780) and cholesterol (catalog no: ab65359) content were analyzed using commercially available colorimetric assays (Abcam, Cambridge, MA) on a Synergy HTX multi‐mode reader (Biotek, Winooski, VT). Glucose concentrations were measured in whole blood using an i‐STAT Blood Analysis System (G Cartridge; Abbott Point of Care, Princeton, NJ). Insulin concentrations were measured in serum using commercially available enzyme‐linked immunosorbent assays (catalog no: EZRMI‐13K; Millipore, Billerica, MA), and insulin resistance was assessed using the homeostatic model assessment of insulin resistance (HOMA‐IR) (Varanoske et al., 2021), where:

$$\begin{array}{c} \text{HOMA}-\mathrm{IR}=\text{Glucose}\;\left(\text{mmol}∙L^{-1}\right)\;\\ \;x\;\text{Insulin}\;\left(\mu IU∙{\mathrm{mL}}^{-1}\right)/22.5. \end{array}$$

### 2.7 Statistical analysis

Normality was assessed for each variable using univariate analysis to quantitatively evaluate skewness and visually inspect box and probability plots. Non‐normally distributed data were log‐transformed. Two‐way ANOVA was used to analyze the main effects of ER (ER vs. AL) and dietary macronutrient content (15% protein, 76% carbohydrate vs. 35% protein, 56% carbohydrate) and their interaction on outcome measures. Post hoc analyses were adjusted by Tukey's multiple comparison procedure when appropriate. Pearson's correlation coefficient was used to determine the strength of associations between lipid kinetic measures and body mass gain. *p* < 0.05 was considered statistically significant. Data were analyzed using JMP®, version 15 and SAS, version 9.4 (SAS Institute Inc., Cary, NC).

## 3 RESULTS

### 3.1 Feed intake

Per the study design, feed intake was greater in the AL groups compared to the ER groups (Table 2, P‐energy <0.001). Macronutrient content did not affect feed intake (*p* = 0.28). There were main effects of energy level (*p* < 0.0001) on daily water consumption and significant energy‐by‐diet interactions (*p* ≤ 0.004) for protein and carbohydrate intake (Table 2). Body water ^2^H enrichments were not different between groups (overall mean, 3.1 ± 0.9%).

**TABLE 2:** Feed, water, and nutrient intake of obese female Zucker rats following 10 weeks of ad libitum or energy restricted feeding with diets higher in carbohydrate or higher in protein content.

	Ad libitum		Energy restriction	*p*‐value			
	HC/LP (*n* = 11)	HP/LC (*n* = 12)	HC/LP (*n* = 12)	HP/LC (*n* = 11)	Energy	Diet	Interaction
Average daily							
Feed intake, g/d	23.8 ± 2.1	24.1 ± 1.4	14.8 ± 0.6	15.7 ± 2.9	<0.001	0.28	0.57
Energy intake, kcal/d	90.6 ± 8.1	91.8 ± 5.2	56.2 ± 2.4	59.8 ± 11.2	<0.001	0.28	0.57
Protein, g/d	3.4 ± 0.3^a^	8.0 ± 0.5^b^	2.1 ± 0.1^c^	5.2 ± 1.0^d^	<0.001	<0.001	<0.001
Carbohydrate, g/d	17.2 ± 1.5^a^	12.8 ± 0.7^b^	10.7 ± 0.5^c^	8.4 ± 1.6^d^	<0.001	<0.001	0.004
Fat, g/d	0.91 ± 0.08	0.92 ± 0.05	0.56 ± 0.02	0.60 ± 0.11	<0.001	0.28	0.57
Water intake, mL/d	20.0 ± 7.3	27.8 ± 6.2	11.9 ± 1.7	15.3 ± 2.3	<0.001	0.09	0.80

*Note*: Values are means ± SD. A two‐way ANOVA was used to analyze the main effects of energy restriction (energy restriction vs. ad libitum) and dietary macronutrient content (15% protein, 76% carbohydrate diet compared to 35% protein, 56% carbohydrate diet) and their interaction on outcome measures. Post hoc analyses were adjusted by Tukey's multiple comparison procedure when appropriate. Values not sharing a superscripted letter are significantly different within each row (*p* < 0.001).

Abbreviations: HC, high carbohydrate; HP, high protein; LC, low carbohydrate; LP, low protein.

### 3.2 Body mass and composition

**FIGURE 1:**
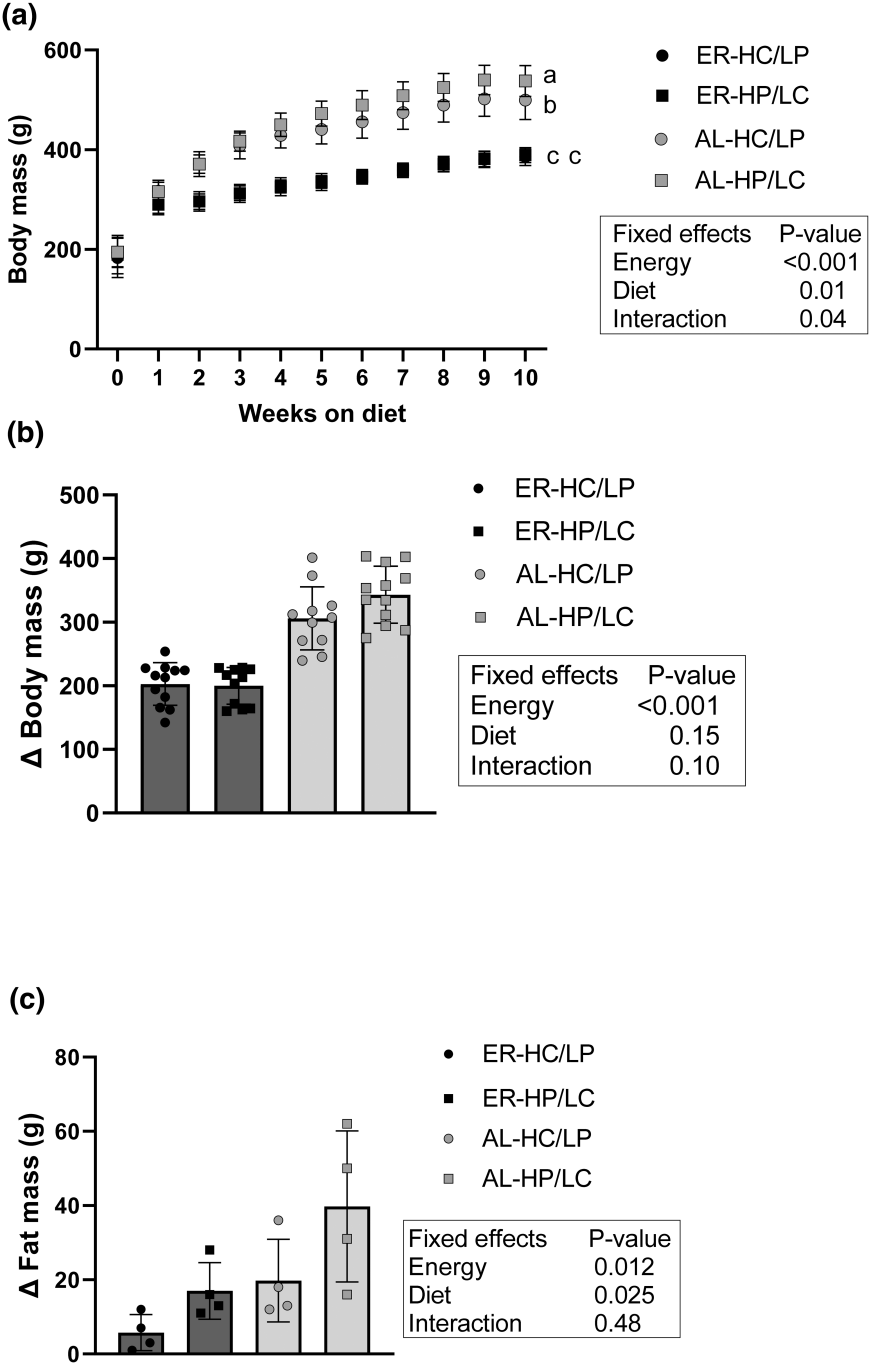
Body mass and composition. (a) Weekly body mass (n = 46), (b) total change in body mass (*n* = 46), and (c) total change in fat mass (*n* = 16) in obese female Zucker rats during 10 weeks of ad libitum or energy restricted feeding with diets higher in carbohydrate or higher in protein content. All data are presented as means ± SD and were analyzed by a two‐way ANOVA. The main effects of energy restriction (energy restriction vs. ad libitum), macronutrient content (15% protein, 76% carbohydrate vs. 35% protein, 56% carbohydrate) and their interactions are depicted by each graph. There was a significant energy‐by‐diet interaction (P‐interaction = 0.037) observed for body mass at 10‐weeks (a). Post hoc analyses were adjusted by Tukey's multiple comparison procedure. Values not sharing a superscripted letter are significantly different. AL, ad libitum; ER, energy restriction; HC, high carbohydrate; HP, high protein; LP, low protein; LC, low carbohydrate.

After the 10‐week study, there was a significant energy‐by‐diet interaction (P‐interaction = 0.037) observed for body mass, AL‐HC/LP (499 ± 39 g) weighed more than both the ER groups (ER‐HC/LP: 386 ± 18 g and ER‐HP/LC: 389 ± 15 g) and AL‐HP/LC (538 ± 31 g) weighed more than all other groups (Figure 1a). For change in body mass (10 week—initial body mass), there was a significant effect of energy (P‐energy <0.001), but no diet or interaction effects (Figure 1b). Specifically, changes in body mass were greater in the AL groups (AL‐HC/LP: 306 ± 50 g, AL‐HP/LC: 343 ± 45 g) compared to the ER groups (ER‐HC/LP: 203 ± 33 g, ER‐HP/LC: 200 ± 29 g). Body fat at the end of the 10‐week study was greater in the AL groups (AL‐HC/LP: 196 ± 12 g, AL‐HP/LC: 213 ± 10 g) compared to the ER groups (ER‐HC/LP: 179 ± 10 g, ER‐HP/LC: 185 ± 7 g; P‐energy = 0.0009) and was greater in the HP/LC groups compared to the HC/LP groups (P‐diet = 0.046). Similarly, changes in fat mass from baseline to the end of study were greater in the AL groups (AL‐HC/LP: 19.6 ± 11.0 g, AL‐HP/LC: 39.8 ± 20.1 g) compared to the ER groups (ER‐HC/LP: 5.8 ± 4.8 g, ER‐HP/LC: 17.1 ± 7.4 g; P‐energy = 0.012) and were greater in the HP/LC groups compared to the HC/LP groups (P‐diet = 0.025; Figure 1c).

### 3.3 Triglyceride synthesis

Fractional TG synthesis was lower in ER‐fed rats than AL‐fed rats in both SAT and VAT (Table 3; main effect of energy, *p* < 0.0001 for both). Hepatic TG synthesis did not differ by energy level or diet (*p* ≥ 0.13) and was greater than 80% in all groups, indicating hepatic TG was nearly completely replaced in the liver over the course of the 7‐day labeling period. TG synthesis in both SAT and VAT were correlated with the amount of body mass gained during the study (*r* = 0.56 and *r* = 0.67, respectively; *p* < 0.0001).

**TABLE 3:** Lipid kinetic measures in obese female Zucker rats following 10 weeks of ad libitum or energy restricted feeding with diets higher in carbohydrate or higher in protein content.

	Ad libitum		Energy restriction		*p*‐value		
	HC/LP (*n* = 11)	HP/LC (*n* = 12)	HC/LP (*n* = 12)	HP/LC (*n* = 11)	Energy	Diet	Interaction
Triglyceride synthesis (%)							
Subcutaneous	29.0 ± 5.7	28.2 ± 5.7	20.8 ± 3.9	18.7 ± 3.6	<0.0001	0.32	0.65
Visceral	29.3 ± 3.8	28.5 ± 4.2	22.8 ± 3.4	21.4 ± 3.4	<0.0001	0.33	0.82
Hepatic	82.1 ± 8.8	80.7 ± 7.6	82.8 ± 7.7	87.0 ± 6.5	0.13	0.54	0.23
De novo palmitate incorporation into triglyceride (%)							
Subcutaneous	15.4 ± 5.2	15.9 ± 3.3	15.9 ± 4.4	13.5 ± 4.5	0.47	0.46	0.26
Visceral	17.6 ± 2.3	16.7 ± 4.4	19.6 ± 8.6	24.5 ± 5.3	0.0057	0.24	0.09
Hepatic	62.3 ± 3.5	57.2 ± 4.9	57.5 ± 11.5	60.0 ± 13.5	0.63	0.55	0.077
De novo cholesterol synthesis (%)							
Subcutaneous	6.2 ± 1.2	4.9 ± 1.3	7.4 ± 2.4	7.1 ± 1.4	0.0019	0.11	0.33
Visceral	6.2 ± 1.0	6.1 ± 2.7	8.3 ± 2.4	8.2 ± 0.9	0.0007	0.86	0.95
Hepatic	15.1 ± 3.0^a^	11.9 ± 3.0^a^	20.9 ± 6.5^b^	23.5 ± 3.5^b^	<0.0001	0.80	0.028

*Note*: Values are means ± SD. A two‐way ANOVA was used to analyze the main effects of energy restriction (energy restriction vs. ad libitum) and dietary macronutrient content (15% protein, 76% carbohydrate diet compared to 35% protein, 56% carbohydrate diet) and their interaction on outcome measures. Post hoc analyses were adjusted by Tukey's multiple comparison procedure when appropriate. Values not sharing a superscripted letter are significantly different within each row (*p* < 0.05).

Abbreviations: HC, high carbohydrate; HP, high protein; LC, low carbohydrate; LP, low protein.

### 3.4 De novo palmitate synthesis

Fractional synthesis of palmitate (i.e., DNL) was lower in ER‐fed rats than AL‐fed rats independent of macronutrient content (main effect of energy, *p* < 0.0001) in SAT, but not in VAT or hepatic tissue. More importantly, ER‐groups, compared to AL groups, had greater relative contribution of de novo palmitate to TG synthesis in VAT (main effect of energy, *p* = 0.0057; Table 3). There were no significant differences in the contribution of DNL to TG in SAT or hepatic tissue.

### 3.5 De novo cholesterol synthesis

In SAT and VAT, de novo cholesterol synthesis was greater for ER‐fed rats than AL‐fed rats, independent of macronutrient content (main effect of energy, *p* = 0.0019 and *p* = 0.0007, respectively). There was a significant interaction effect (P‐interaction = 0.028) for hepatic de novo cholesterol synthesis and post hoc comparisons indicated ER‐HC/LP and ER‐HP/LC had greater cholesterol synthesis compared to AL‐HC/LP and AL‐HP/LC (Table 3).

### 3.6 Metabolic biomarkers

Insulin and glucose concentrations and HOMA‐IR were high in all groups (Table 4). Insulin concentrations did not differ by energy intake or dietary macronutrient content, while glucose concentrations were higher in the ER than AL groups (main effect of energy, *p* = 0.036). HOMA‐IR did not differ by energy intake or dietary macronutrient content. Serum TG concentrations were lower in the ER groups compared to the AL groups (main effect of energy, *p* = 0.0003), but were not affected by macronutrient content. Serum free fatty acid concentrations were lower in the HC/LP groups compared to the HP/LC groups (main effect of diet, *p* = 0.018). Serum cholesterol concentrations were lower in the AL groups compared to the ER groups (main effect of energy, *p* = 0.0035) and HP/LC groups compared to HC/LP groups (main effect of diet, *p* = 0.0064).

**TABLE 4:** Blood and liver metabolic measures in obese female Zucker rats following 10 weeks of ad libitum or energy restricted feeding with diets higher in carbohydrate or higher in protein content.

	Ad libitum				Energy restricted				*p*‐value		
	*n*	HC/LP	*n*	HP/LC	*n*	HC/LP	*n*	HP/LC	Energy	Diet	Interaction
Blood											
Triglyceride (mg/dL)*	6	92 [63, 136]	6	156 [78, 312]	6	52 [40, 68]	6	58 [42, 81]	0.0003	0.085	0.26
Cholesterol (mg/dL)	10	125 ± 11	10	111 ± 7	10	131 ± 13	10	125 ± 10	0.0035	0.0064	0.23
Free fatty acids (μM)*	8	3.67 [3.20, 4.20]	8	5.16 [3.51, 7.59]	8	3.72 [3.22, 4.28]	8	4.34 [3.62, 5.20]	0.42	0.018	0.35
Insulin (pmol/L)	9	444 ± 177	9	332 ± 189	7	364 ± 244	10	353 ± 177	0.66	0.36	0.45
Glucose (mmol/L)	11	11.7 ± 2.7	12	8.9 ± 1.2	12	12.0 ± 3.5	11	12.6 ± 4.4	0.036	0.26	0.071
HOMA‐IR	9	37.0 ± 15.6	9	22.4 ± 13.0	7	28.1 ± 20.8	10	30.3 ± 14.8	0.93	0.26	0.13
Liver											
TG concentration (nmol/mg)	5	15.5 ± 4.9^a,b^	5	15.8 ± 7.5^a,b^	5	27.0 ± 10.2^a^	5	7.1 ± 6.9^b^	0.69	0.011	0.0091
TG synthesized (nmol/mg)	5	12.8 ± 4.5^a,b^	5	13.5 ± 7.2^a,b^	5	23.0 ± 9.0^a^	5	6.2 ± 5.9^b^	0.66	0.019	0.012
Cholesterol (mg/g)	7	1.43 ± 0.29	7	1.33 ± 0.31	7	1.66 ± 0.22	7	1.47 ± 0.15	0.065	0.13	0.60
Cholesterol synthesized (mg/g)	7	0.21 ± 0.06	7	0.17 ± 0.04	7	0.36 ± 0.13	7	0.36 ± 0.05	<0.0001	0.50	0.47

*Note*: Normally distributed data are presented as means ± SD and log‐transformed data as geometric means with upper and lower 95% confidence levels. A two‐way ANOVA was used to analyze the main effects of energy restriction (energy restriction vs. ad libitum) and dietary macronutrient content (15% protein, 76% carbohydrate diet compared to 35% protein, 56% carbohydrate diet) and their interaction on outcome measures. Post hoc analyses were adjusted by Tukey's multiple comparison procedure when appropriate. Two outliers (both in the ER‐HC/LP group) were identified in the insulin concentration (1087 and 1989 pmol/L) and HOMA‐IR (128 and 184) data and removed from the analysis. Values not sharing a superscripted letter are significantly different within each row (*p* < 0.05).

Abbreviations: HC, high carbohydrate; HOMA‐IR, homeostatic model assessment of insulin resistance; HP, high protein; LC, low carbohydrate; LP, low protein; TG, triglyceride.

* Indicates the log of the value was used for statistical analysis.

There was an interaction effect for hepatic TG concentrations (P‐interaction = 0.0091) and absolute hepatic TG synthesis (P‐interaction = 0.012), with post hoc comparisons indicating ER‐HC/LP had higher TG concentrations (P‐adjusted = 0.0073) and absolute hepatic TG synthesis (P‐adjusted = 0.0068) than ER‐HP/LC. Hepatic cholesterol concentrations (main effect of energy, *p* = 0.065) and absolute hepatic cholesterol synthesis (main effect of energy, *p* < 0.0001) were greater in the ER groups compared to the AL groups.

## 4 DISCUSSION

Obesity contributes to AT remodeling and a pro‐inflammatory and insulin‐resistant state, which contributes to ectopic lipid deposition, increased hepatic de novo lipogenesis, excess VAT accumulation, and altered SAT TG turnover (Eissing et al., 2013; Lee et al., 2010; Longo et al., 2019; Nouws et al., 2019). Previous research suggests lower adipose tissue TG synthesis is a marker of impaired TG storage capacity, which may contribute to dyslipidemia, ectopic lipid accumulation, and insulin resistance (Allister et al., 2015; Arner et al., 2011; Ravussin & Smith, 2002; Tuvdendorj et al., 2013). Dietary ER and macronutrient manipulation are approaches commonly employed to reduce body weight and improve metabolic outcomes in humans and rodents (Morens et al., 2006; Noakes et al., 2005). In the current study, the Zucker rat (fa/fa) is used as a model of obesity and is characterized by a mutated leptin receptor. These animals display hyperphagia, obesity, and insulin resistance.

In the present study, animals were relatively weight stable during the last 7 days of the study (i.e., heavy water dosing period; Figure 1), indicating similar rates of TG synthesis to rates of lipolysis to maintain weight stability during this timeframe. However, there were differences in fat accumulation between energy intake levels (i.e., AL vs. ER) and diet groups (i.e., HC/LP vs. HP/LC) over the course of the 10‐week study likely indicating differences in lipolysis rates, in addition to TG synthesis rates, over the duration of the study. With reduced TG synthesis leading to less fat accumulation in the ER groups, increased lipolysis rates were likely driving reduced fat accumulation in the HC/LP groups. This is supported by our finding that the HC/LP groups had greater hepatic TG content. However, greater lipolysis in the HC/LP group is not supported by reduced concentrations of fasted FFA in the HC/LP compared to HP/LC group. Greater FFA concentrations in the HP/LC group is reflective of the fasting state only and could be due to increased fat mass in the HP/LC group. Increased adipose tissue TG retention in the HP/LC group may be indicative of improved lipid storage capacity due to reductions in SAT and VAT lipolysis and serve a protective function by decreasing the mobilization of lipids to peripheral organs.

We observed no effects of ER on fractional TG synthesis rates in the liver. Hepatic fractional TG synthesis ranged from 80% to 87%, indicating liver TG was almost completely replaced within 7‐days in this model of obesity (Turner et al., 2003). Furthermore, there were no effects of ER on hepatic DNL rates or intrahepatic TGs. This is in agreement with a study providing a 30% energy restricted diet to 9‐week‐old C57BL/6J male mice, which showed no differences in daily hepatic DNL compared to ad libitum fed controls (Bruss et al., 2009). However, our findings are contrary to previous studies showing 10% weight loss due to ER reduces hepatic DNL and intrahepatic TG content in humans (Smith et al., 2020) and 40% energy deficit for 16 weeks decreases intrahepatic TG and hepatic DNL‐related gene expression (but not DNL‐related protein expression) in 12‐week‐old, male Sprague Dawley rats (Margolis et al., 2016). There are methodological (i.e., ER leading to weight loss vs. attenuated weight gain) and model (i.e., inclusion of older male Sprague Dawley rats vs. younger, female Zucker rats) differences that may explain these discrepant findings for hepatic DNL following ER.

Importantly, in the current study, absolute hepatic TG synthesis and intrahepatic TG content were reduced in energy‐restricted animals eating more protein and less carbohydrate compared to energy‐restricted animals eating more carbohydrate and less protein. These findings are in general agreement with Margolis et al., who reported reductions in intrahepatic TG content with higher protein (HP) feeding (32% protein, 46% carbohydrate) compared to higher carbohydrate feeding (10% protein, 70% carbohydrate), independent of energy intake, in male Sprague Dawley rats (Margolis et al., 2016). Taken together, our data indicate macronutrient content of the diet (i.e., lower carbohydrate (LC) intake with replacement by protein), in addition to energy‐restriction, is critical for improving metabolic health in this genetically driven model of obesity.

In the present study, energy restricted groups had higher rates of de novo cholesterol biosynthesis in all three measured depots (SAT, VAT, and liver) and greater serum cholesterol concentrations. This result was surprising given fasting and ER typically reduce circulating cholesterol concentrations (Di Buono et al., 1999; Hirowatari et al., 2017; Triscari et al., 1980). However, we speculate that this finding may be due to a “meal‐feeding” effect in the energy‐restricted animals. Energy‐restricted animals were fed each morning, likely consuming all the food provided in a relatively short time span (i.e., meal‐feeding), instead of grazing throughout the day like the ad libitum animals. In humans, increasing the number of eating occasions each day reduces blood cholesterol levels (St‐Onge et al., 2017). For instance, adults eating 17 snacks/day versus 3 meals/day, had decreased total cholesterol, LDL‐cholesterol, and insulin concentrations (Jenkins et al., 1989), but no difference in blood glucose or mevalonic acid excretion (a marker of cholesterol synthesis in the liver) (Jenkins et al., 1995). In a study of energy‐restricted, meal fed obese Zucker rats, peak circulating cholesterol concentration was greater and glucose concentrations were higher at all timepoints throughout the day compared to ad libitum control animals (Lanza‐Jacoby et al., 1986). It has been suggested that greater postprandial exposure to insulin and/or glucose following fewer, but larger, meals may drive the increase in cholesterol concentrations. We have observed in human subjects that synthesis of plasma cholesterol, measured by heavy water administration, is highly correlated with 24‐h insulin and glucose concentrations (Smith G, Hellerstein M, Klein S, personal communication, March 6, 2023). In the current study, fasting glucose concentrations were higher in the energy‐restricted group compared to the ad libitum group (P‐energy = 0.036), and glucose was associated with VAT cholesterol synthesis (*r* = 0.29, *p* = 0.049) and serum cholesterol concentrations (*r* = 0.32, *p* = 0.045). However, glucose was not associated with liver or SAT cholesterol synthesis or hepatic cholesterol concentrations. Insulin concentrations and HOMA‐IR were not associated with liver, SAT, or VAT cholesterol synthesis, serum cholesterol concentrations, or hepatic cholesterol concentrations. The correlational data should be interpreted carefully as it only represents the postabsorptive state and energy‐restricted animals had not eaten since the previous morning (i.e., ~24 h without food) compared to the ad libitum animals who were fasted overnight (i.e., ~12 h without food).

Study limitations include a lack of adipogenesis measures that could further elucidate the effects of ER and dietary protein content on AT expansion (hypertrophic vs. hyperplasia). Calorie restriction changes eating behaviors where ER rats tend to eat all the feed provided very rapidly. Gorging behaviors are documented to induce changes in genes that promote lipid storage, which is an inherent limitation of animal trials involving ER (Kliewer et al., 2015). Previous studies have demonstrated that leptin deficiency (*ob/ob* mice) significantly increases lipogenic flux independent of hyperphagia and hyperinsulinemia (Turner et al., 2007). Therefore, use of leptin receptor knockout animals as a model of obesity has limited generalizability to polygenic rodents with diet‐induced obesity, which are more representative of human obesity, and wildtype rodents with normal lipid flux, especially since a lean control group was not included in the current study. Furthermore, since only female Zucker rats were included in the current study, the effects of ER and diet composition on lipid flux in male Zucker rats are unknown. Another study limitation is the uncertainty of the original biosynthetic site of TG measured in different adipose depots, which represents the TG storage site. Importantly, TG deposition in AT far exceeds hepatic TG export quantitatively (Smith et al., 2020). Finally, limiting the energy restricted groups to 60% of the feed consumed by the ad libitum groups did not induce weight loss in this genetic model of obesity, but merely slowed the rate of weight gain. A major strength of the study is the use of ^2^H_2_O to measure in vivo lipid flux in physiologically relevant depots (SAT, VAT, and liver).

## 5 CONCLUSION

In conclusion, ER alone did not improve lipid kinetics or hepatic TG content, and unexpectedly increased hepatic and AT (SAT and VAT) cholesterol synthesis in female Zucker rats. Replacing dietary carbohydrate (from 76% to 56%) with protein (from 15% to 35%) led to increased AT stores and reductions in hepatic absolute TG synthesis and TG content, particularly in energy‐restricted animals. Increased AT TG retention with HP/LC feeding may reflect improved lipid storage capacity and serve a protective function by decreasing lipid deposition in peripheral organs in this genetically driven rodent model of obesity.

## AUTHOR CONTRIBUTIONS

Stephen R. Hennigar, James P. McClung, Mahalakshmi Shankaran, William J. Evans, Marc K. Hellerstein, Stefan M. Pasiakos, and Claire E. Berryman designed the research; Stephen R. Hennigar, Mahalakshmi Shankaran, Edna Nyangau, Tyler J. Field, Alyssa M. Kelley, Bradley J. Anderson, and Claire E. Berryman conducted the research; M. Alan Dawson, Stephen R. Hennigar, Mahalakshmi Shankaran, William J. Evans, Marc K. Hellerstein, and Claire E. Berryman analyzed the data; M. Alan Dawson and Claire E. Berryman wrote the paper; Claire E. Berryman had primary responsibility for final content. All authors read and approved the final manuscript. The authors have no conflicts of interest to declare.

## FUNDING INFORMATION

Funded by the US Army Medical Research and Development Command and appointment to the US Army Research Institute of Environmental Medicine administered by the Oak Ridge Institute for Science and Education through an interagency agreement between the US Department of Energy and the US Army Medical Research and Development Command.

## ETHICS STATEMENT

This study was approved by the Institutional Animal Care and Use Committee at the US Army Research Institute of Environmental Medicine, which is accredited by the Association for Assessment and Accreditation of Laboratory Animal Care International.

## Data Availability

The data that support the findings of this study are available on request from the corresponding author.
